# The association between quality of connections and diagnostic accuracy in student-generated concept maps for clinical reasoning education with virtual patients

**DOI:** 10.3205/zma001643

**Published:** 2023-09-15

**Authors:** Andrzej A. Kononowicz, Dario Torre, Stanisław Górski, Michał Nowakowski, Inga Hege

**Affiliations:** 1 Jagiellonian University Medical College, Department of Bioinformatics and Telemedicine, Kraków, Poland; 2 University of Central Florida College of Medicine, Department of Medical Education, Orlando (FL), USA; 3 Jagiellonian University Medical College, Department of Medical Education, Center for Innovative Medical Education, Kraków, Poland; 4 Jagiellonian University Medical College, 2nd Department of General Surgery, Kraków, Poland; 5 University of Augsburg, Medical Education Sciences, Augsburg, Germany

**Keywords:** computer-assisted instruction, virtual patients, concept maps, clinical reasoning

## Abstract

**Objectives:**

Concept maps are a learning tool that fosters clinical reasoning skills in healthcare education. They can be developed by students in combination with virtual patients to create a visual representation of the clinical reasoning process while solving a case. However, in order to optimize feedback, there is a need to better understand the role of connections between concepts in student-generated maps. Therefore, in this study we investigated whether the quality of these connections is indicative of diagnostic accuracy.

**Methods:**

We analyzed 40 concept maps created by fifth-year medical students in the context of four virtual patients with commonly encountered diagnoses. Half of the maps were created by students who made a correct diagnosis on the first attempt; the other half were created by students who made an error in their first diagnosis. The connections in the maps were rated by two reviewers using a relational scoring system. Analysis of covariance was employed to examine the difference in mean connection scores among groups while controlling for the number of connections.

**Results:**

There were no differences between the groups in the number of concepts or connections in the maps; however, maps made by students who made a correct first diagnosis had higher scores for the quality of connections than those created by students who made an incorrect first diagnosis (12.13 vs 9.09; p=0.03). We also observed students’ general reluctance to use connections in their concept maps.

**Conclusion:**

Our results suggest that the quality, not the quantity, of connections in concept maps is indicative of their diagnostic accuracy.

## Zusammenfassung

**Zielsetzung:**

Concept Maps sind ein Werkzeug, das die Fähigkeiten zur klinischen Entscheidungsfindung in der Ausbildung in den Gesundheitsberufen fördert. Sie können von Studierenden in Kombination mit virtuellen Patient*innen erstellt werden, um so den klinischen Denkprozess bei der Lösung eines Falles zu visualisieren. Um das Feedback zu optimieren, ist es jedoch notwendig, die Bedeutung der Verbindungen zwischen den Konzepten in den von den Studierenden erstellten Maps besser zu verstehen. Daher haben wir in dieser Studie untersucht, ob die Qualität von Verbindungen ein Indikator für die Diagnosegenauigkeit ist.

**Methoden:**

Wir analysierten 40 Concept Maps, die von Medizinstudierenden im fünften Studienjahr während der Bearbeitung von vier virtuellen Patient*innen mit häufigen Erkrankungen erstellt wurden. Die eine Hälfte der Concept Maps wurde von Studierenden erstellt, die beim ersten Versuch eine korrekte Diagnose stellten, die andere von Studierenden, die bei ihrer ersten Diagnose einen Fehler machten. Die Verbindungen in den Maps wurden von zwei Reviewer*innen auf Basis eines relationalen Punktesystems bewertet. Mithilfe einer Kovarianzanalyse untersuchten wir den Unterschied der Durchschnittsscores für die Verbindungen zwischen den Gruppen unter Kontrollierung der Anzahl der Verbindungen.

**Ergebnisse:**

Es gab keine Unterschiede zwischen den Gruppen bezüglich Anzahl der Konzepte oder Verbindungen in den Maps. Allerdings hatten die Maps der Studierenden, mit korrekter Erstdiagnose, eine höhere Qualität der Verbindungen als die Maps mit falscher Erstdiagnose (12,13 vs. 9,09; p=0,03). Wir beobachteten auch, dass die Studierenden generell wenige Verbindungen in ihren Concept Maps zogen.

**Schlussfolgerung:**

Unsere Ergebnisse deuten darauf hin, dass die Qualität, nicht die Quantität, der Verbindungen in Concept Maps für die Diagnosegenauigkeit ausschlaggebend ist.

## 1. Introduction

Concept maps are a graphic representation of learners’ knowledge organization that entails a series of concepts connected by linking words [1]. They can promote meaningful learning, enable educators to provide feedback to learners, and can be used for assessment purposes [2], [3]. Through the use of concept maps, students may foster their knowledge organization and facilitate problem representation, both of which are critical components of the clinical reasoning process [4]. Moreover, concept maps give students an opportunity to organize information related to complex clinical problems. They promote formation of illness scripts by enabling students to discern patterns of clinical features that are characteristic of particular diseases [5]. Teachers can analyze concept maps to identify learners’ misunderstandings, knowledge gaps and errors [6].

Virtual patients (VPs) provide a safe learning environment in which learners take the role of a healthcare professional to practice clinical reasoning [7], [8]. By following a patient story, students reveal new clinical findings and make decisions regarding the diagnostic and management process. This safe environment gives students the opportunity to learn from and receive automated feedback about their errors. Empirical studies have shown that VPs support acquisition of clinical reasoning abilities [8], [9].

To improve the training of clinical reasoning and to benefit from what concept maps offer, we integrated a concept-mapping approach in which learners are prompted to visualize their clinical reasoning process while solving VP cases [10]. Concept maps provide teachers with an opportunity to evaluate each student’s understanding, knowledge organization, and processing of clinical information.

A previous study showed no correlation between diagnostic accuracy and the number of connections made by medical students in their concept maps while working on VPs [11]. However, the impact of accurate and valid connections on diagnostic accuracy has not previously been evaluated. Therefore, the purpose of our study was to determine whether the quality of connections among concepts in clinical reasoning maps developed in a VP environment was associated with students’ diagnostic accuracy.

Ausubel’s assimilation theory provided the theoretical framework for this study [12]. Ausubel makes a distinction between rote and meaningful learning: rote learning is less related to prior knowledge because it does not require conscious effort to connect new information to existing concepts; meaningful learning involves the development of relationships among concepts in which the meaning of the relatedness of words is of great importance in the development of new knowledge structures, new meanings and new reasoning. Drawing connections between concepts is critical to fostering propositional knowledge, which contains the meanings of the relationships between concepts. Therefore, in our study, we hypothesized that students who developed high-quality connections in their concept maps would perform better than those who created low-quality connections.

## 2. Methods

### 2.1. Participants

We analyzed concept maps created by fifth-year medical students enrolled at the medical school of Jagiellonian University Medical College in Kraków, Poland who participated in the Laboratory Training of Clinical Skills (LTCS) course in the years 2017-2019.

### 2.2. Data collection

**Figure 1:**
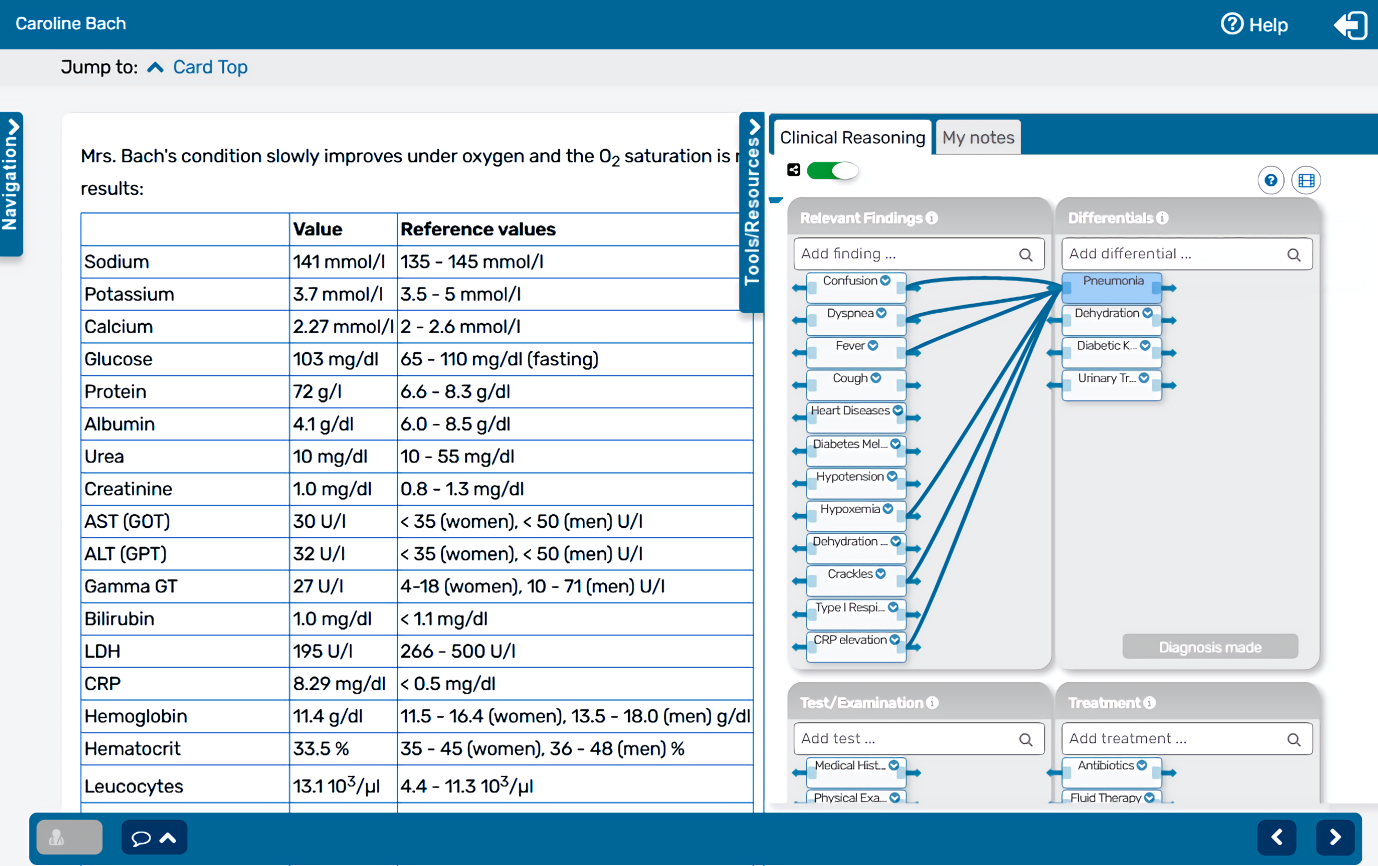
Screenshot of an exemplary VP with a concept map for clinical reasoning

As part of the LTCS course, each student had to complete 16 VPs from a pool of open-access cases [https://crt.casus.net]. Students accessed the cases using an individual login code as a form of self-study in preparation for their classes in the course. The VPs included a variety of key presenting complaints and demographics. Part of the students’ assignment was to create a concept map for each VP using the concept mapping tool developed by the authors [10] and integrated with the CASUS VP system (a sample is presented in figure 1 (Fig. 1)). These maps linked symptoms, differential diagnoses, diagnostic workup and treatment. At a certain point in the VP scenario, students were asked to submit a final diagnosis. If their final diagnosis was incorrect, they received feedback and could either resubmit the final diagnosis or receive the correct final diagnosis from the system. Prior to working on the VPs, students were introduced to the tool by watching a short video tutorial displayed upon the first use of the tool.

Out of the 16 VPs solved by the students, we selected four for in-depth analysis. We applied the following criteria for selecting the maps for our analysis: VPs included relevant content for senior medical students; the cases depicted commonly encountered diagnoses across different organ systems (gastrointestinal, cardiovascular and respiratory); the VPs had different difficulty levels and gender (2 males and 2 females). Based on these criteria, we selected maps that covered a range of topics: enterocolitis, pneumonia, ulcerative colitis, and aortic valve stenosis.

Next, we divided the student-authored concept maps for these four VPs into two groups based on whether the student’s first diagnosis was correct or incorrect. In order to evaluate the impact of the quality of connections, we stratified the groups according to the number of connections in the maps (low range: 1-10 connections; medium: 11-15; and high: 16-30). We selected 40 maps for analysis (20 in each group).

Finally, two authors (DT, IH) rated the quality of these connections based on a rubric we developed that was derived from the relational scoring literature [2]. In contrast to structural scoring, relational scoring focuses on the quality and importance of each individual link and not on the overall organization, hierarchy and cross links of the map [13]. Raters assigned points to each connection depending on whether they were valid and helpful (2 points), partially valid (1 point) or invalid (0 points). Details of the rubric used in this study are presented in attachment 1 . Cohen’s Kappa was calculated as a measure of inter-rater reliability in judging connection quality. In the case of divergent judgments, a post-hoc consensus on the final score was achieved by discussion among the raters. Points for all connections were added together to obtain the overall score for a given map.

### 2.3. Statistical analysis

Statistical calculations were performed in Statistica 13.3 [https://www.tibco.com/] and R 4.1.2 [https://www.r-project.org/] with level of significance set to α=0.05. We compared the mean number of concepts and connections across groups with the Student’s t-test and verified the normal distribution requirement using the Shapiro-Wilk test. Analysis of covariance (ANCOVA) was employed to examine the difference in mean connection scores among groups while controlling for the number of connections. Cohen’s Kappa was calculated as a measure of inter-rater reliability in judging the connection validity prior to reaching a consensus on the final rating.

### 2.4. Ethics

The study was approved by the ethics committee of Jagiellonian University (No. 122.6120.116.2016).

## 3. Results

In total, 222 fifth-year medical students enrolled in the LTCS course. The students created 3382 maps that included a final diagnosis. Of this number, 623 maps (18%) also included connections. For the four VPs selected for analysis, there were 176 maps, of which n=112 led to a correct diagnosis on the first attempt (no error group), while n=64 led to an incorrect first diagnosis (error group).

We selected for analysis a sample of 40 maps (20 with and 20 without errors). These maps were created by 32 different students: eight students contributed two maps each; 24 students contributed one map each. The two raters reached a good level of agreement regarding their connection ratings on the first attempt (Kappa=0.85 CI=[0.79; 0.9]).

The students’ maps in the error group were not different in terms of the number of concepts and connections compared to the non-error group (see table 1 (Tab. 1)) (p>0.05). However, the connection score of maps in the non-error group was significantly higher than that in the error group (p=0.03).

## 4. Discussion

Our study showed that students who made errors in formulating the final diagnosis created less accurate connections in their maps compared to those who did not make errors. The results of this study are supported by the theoretical tenets of assimilation theory [12], which argue that meaningful learning is created by propositional knowledge, and accurate connections between concepts indicate deep learning and understanding. We have demonstrated in our study that faulty or incorrect connections between concepts are related to diagnostic errors in a simulated environment.

The implication of this finding is that teachers should evaluate the quality of connections in concept maps as an indicator of knowledge organization and learners’ understanding. A previous study showed that simple automatic metrics of students' performance based merely on network structure – for instance, on the number of concepts or connections in concept maps – are not helpful in generating feedback or for assessment [11]. Other studies recommend using network properties such as graph density as an indicator of concept map quality [14]. Our study suggests that teachers should focus their efforts on evaluating the validity of connections in order to assess learners’ knowledge organization in clinical reasoning. This step should not be omitted in assessment of clinical reasoning based on students’ concept maps.

The addition of meaningful connections shows good knowledge organization and provides excellent opportunities for feedback [15]. The use of concept maps in clinical reasoning has been shown to have advantages over verbal-text descriptions [16]. However, we should also consider that high-density maps (i.e., a high number of connections in relation to the number of concepts) might be difficult to assess due to the multitude of connections, therefore the cognitive load is higher [17].

Even though the students in our study were explicitly instructed to draw connections in their maps, most of them (82%) did not do so. The reasons for this are unclear to us, but it is possible that our guidance in the video tutorial was insufficient. This aligns with findings from former research that introductory sessions that include feedback are an important step in implementing concept maps in curricula [3]. Previous research has also shown that peer feedback improves learning outcomes, whereas using concept maps without feedback is ineffective [18]. More research is needed to explore these questions.

The study has limitations. First, we included a group of senior medical students from just one institution, which may limit its generalizability to other medical schools. Second, we are aware of case specificity in clinical reasoning, therefore the results of this study may not be applicable to other cases, content, or contexts [19], [20]. However, we sampled common complaints across different organ systems. Third, our sample size of maps was relatively small, yet it had enough power to detect a statistically significant difference.

## 5. Conclusions

This study demonstrates that the quality of connections in student-generated clinical reasoning concept maps in the context of a VP environment is related to diagnostic accuracy. The implications for teachers is that they should consider connection quality in the assessment and feedback of these maps. More research is needed to guide students on how to use connections in these maps and to support teachers by suggesting effective methods of providing feedback to students about faulty connections.

## Acknowledgements and funding

We would like to thank Mr. Andrzej Stanisz for guidance in statistical analyses. The study was approved by the ethics committee of Jagiellonian University (No. 122.6120.116.2016) and supported by internal university grants: K/ZDS/006367 and N41/DBS/000720. 

## Competing interests

The authors declare that they have no competing interests.

## Supplementary Material

Table A1: Rubric for assessing the quality of the connections

## Figures and Tables

**Table 1:**
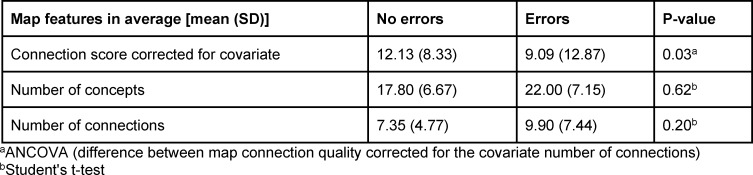
Comparison of student map features across no-error and error groups
