# Supplementary material for: The association between quality of connections and diagnostic accuracy in student-generated concept maps for clinical reasoning education with virtual patients
Source: GMS J Med Educ. 2023 Sep 15;40(5):Doc61. doi: 10.3205/zma001643 (PMC10594037; doi:10.3205/zma001643)
Supplement: Table A1: Rubric for assessing the quality of the connections [file JME-40-61-s-001.pdf]

Attachment 1: Table A1: Rubric for assessing the quality of the connections

**Connection rating**	**Comment**	**Score**
Valid and helpful	The connection is valid, and the relationship between concepts can be clearly established; it is direct (without intermediate steps) e.g. Cough-Pneumonia.	2
Partially valid	• The connection is valid but the meaning of the relationship cannot be clearly established.<br>• Connection is correct, but at least one concept is inaccurate or not part of the differential.<br>• Everything is correct, but the connection is not important for the process.<br>• The relationship can be established, is plausible but is not direct (ABG and Heart failure; LVH and MI); there are several intermediate steps.	1
Invalid	Connection is incorrect; one of the concept is incorrect and/or is not part of the differential, has no meaning, is incorrectly labeled.	0

Attachment 1 to Kononowicz AA, Torre D, Górski S, Nowakowski M, Hege I. *The association between quality of connections and diagnostic accuracy in student-generated concept maps for clinical reasoning education with virtual patients.* GMS J Med Educ. 2023;40(5):Doc61. DOI: 10.3205/zma001643
